# Enhanced Kidney Targeting and Distribution of Tubuloids During Normothermic Perfusion

**DOI:** 10.3389/ti.2025.14747

**Published:** 2025-09-05

**Authors:** Enrique Montagud-Marrahi, Adriana Rodriguez-Gonzalo, Rubén López-Aladid, Yosu Luque, Ruben Rabadán-Ros, Elena Cuadrado-Payan, Elisenda Bañón-Maneus, Jordi Rovira, Marta Lazo-Rodríguez, Oriol Aguilà, Carolt Arana, Ainhoa García-Busquets, Natalia Hierro, Thomas Prudhomme, Mireia Musquera, Yun Xia, Fritz Diekmann, Josep M. Campistol, Maria José Ramírez-Bajo

**Affiliations:** ^1^ Kidney Transplant Unit, Nephrology and Kidney Transplantation Department, Hospital Clinic of Barcelona, Barcelona, Spain; ^2^ Laboratori Experimental de Nefrologia i Trasplantament (LENIT), Fundació per a la Recerca Biomèdica - Institut d’Investigacions Biomèdiques August Pi i Sunyer, Barcelona, Spain; ^3^ Red de Investigación Cooperativa Orientada a Resultados en Salud (RICORS 2040), Madrid, Spain; ^4^ Sorbonne Université - Inserm UMRS_1155, Paris, France; ^5^ Assistance Publique Hopitaux de Paris. Soins Intensifs Nephrologiques et Rein Aigu, Departement de Nephrologie, Hopital Tenon, Paris, France; ^6^ Group of Metabolism and Genetic Regulation of Disease, UCAM HiTech Sport & Health Innovation Hub, Universidad Católica de Murcia, Murcia, Spain; ^7^ Nantes Université, CHU Nantes, INSERM, Centre for Research in Transplantation and Translational Immunology, UMR 1064, Nantes, France; ^8^ Urology Department, Hospital Clinic of Barcelona, Barcelona, Spain; ^9^ Lee Kong Chian School of Medicine, Nanyang Technological University, Singapore, Singapore

**Keywords:** tubuloids, kidney regeneration, transplantation, cell therapy, normothermic perfusion

## Abstract

Tubuloids have become a promising tool for modeling and regenerating kidney disease, although their ability for integration and regeneration *in vivo* is not well documented. Here, we established, characterized, and compared human tubuloids using two optimized protocols: one involving prior isolation of tubular cells (Crude tubuloids) and the other involving prior isolation of proximal tubular cells (F4 tubuloids). Next, healthy rat-derived tubuloids were established using this protocol. Finally, we compared two strategies for delivering GFP tubuloids to a kidney host: 1) subcapsular/intracortical injection and 2) tubuloid infusion during normothermic preservation in a rat transplantation model and a discarded human kidney. F4 tubuloids achieved a higher level of differentiation state compared to Crude tubuloids. When analyzing tubuloid delivery to the kidney, normothermic perfusion was found to be more efficient than *in vivo* injection. Moreover, fully developed tubules were observed in the host parenchyma at 1 week and 1 month after infusion during normothermic perfusion represent a potential strategy to enhance the translatability of kidney regenerative therapies into clinical practice to condition kidney grafts and to treat kidney diseases.

## Graphical Abstract

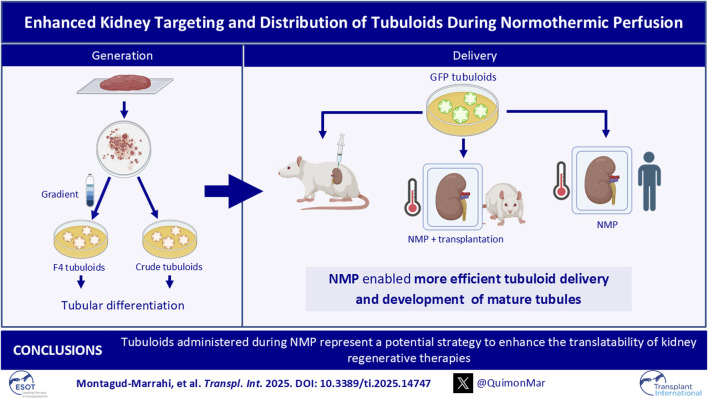

## Introduction

Among the different models of kidney organoids that have been proposed to date, tubuloids have been recently developed as a new model of kidney organoids. Kidney tubuloids consist of a more mature cellular structure of kidney tubular cells that mimic kidney tubules in a three-dimensional distribution and with high precision [1–3]. An important advantage of these constructs is that they can be obtained from adult kidney tissue and they are associated with less technical complexity compared to kidney organoids, since they are developed *in vitro* from cells extracted from kidney tissue (biopsies or nephrectomies) or directly from urine [2].

In addition to *in vitro* disease modeling, tubuloids offer a potential strategy for renal regenerative medicine, although studies on their *in vivo* application have yet to be performed. Previous experiences have shown the successful integration of kidney organoids *in vivo* using different techniques, although the majority of these only achieve a local integration and are difficult to translate into clinical practice [4, 5]. Therefore, recent studies have focused on new approaches to efficiently reach the target organ with the desired treatment. Among them, *ex vivo* normothermic perfusion (NMP) of solid organs has arisen as one of the most promising platforms for organ-specific treatment delivery [6–10]. This system creates a platform for administering and delivering regenerative therapies to the target organ in a controlled and efficient manner [6].

Here, we describe two adapted protocols for obtaining human and rat tubuloids performing a previous purification of tubular cells to explore tubuloid infusion during kidney NMP as a strategy for efficiently delivering tubuloids to the kidney that may be translated into clinical practice.

## Materials and Methods

### Ethics

The animal studies were approved by and conducted according to the guidelines of the local animal ethics committee (Comité Ètic d’Experimentació Animal, CEEA, Decret 214/97, Catalonia, Spain) and the Research Ethics Committee of our center (Comité d’Ètica de la Investigació amb medicaments, Hospital Clinic de Barcelona, HCB/2021/0489).

Experiments performed with human samples were approved by the Research Ethics Committee of our center (Comité d’Ètica de la Investigació amb medicaments, Hospital Clinic de Barcelona, HCB/2021/0490). Written informed consent was obtained from the individuals (for kidney samples and human tubuloid development) and the donors’ next of kin (for discarded human kidney experiments) for the publication of any potentially identifiable images or data included in this article.

### Human Tubuloid Culture

Human kidney tissue for tubuloid culture was collected after nephrectomy from areas of healthy parenchyma from patients with kidney neoplasms but preserved kidney function.

**FIGURE 1:**
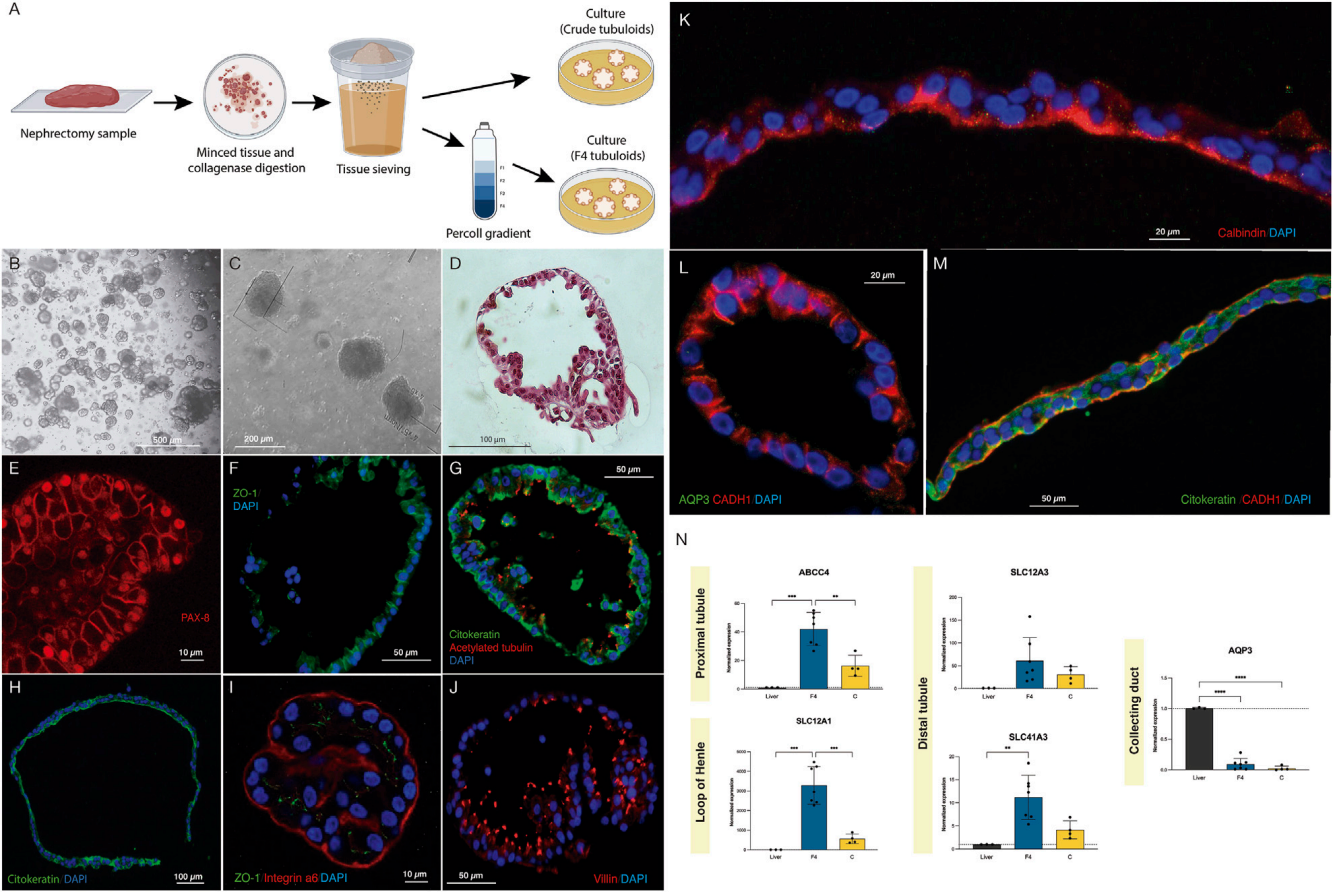
Crude and F4 human kidney tubuloids show typical tubular polarity and specific markers of different nephron segments. **(A)** Methods for obtaining Crude and F4 tubuloids. **(B,C)** Representative images from optical microscopy of human F4 **(B)** and Crude tubuloids **(C)** showing spheroid morphology (obtained from n = 7 independent kidney tissue donors). **(D)** Paraffin sections with HE-staining of a fully-developed F4 human kidney tubuloid with a single cell layer and a spheroid shape (n = 7 independent kidney tissue donors). **(E)** PAX-8 immunofluorescence in a Crude human tubuloid. **(F)** immunofluorescence for ZO-1 in an F4 tubuloid. **(G)** Immunofluorescence for pan-cytokeratin and acetylated tubulin in an F4 tubuloid. **(H)** Immunofluorescence for pan-cytokeratin in a Crude tubuloid. **(I)** Immunofluorescence for Integrin a6 and ZO-1 in an F4 tubuloid. **(J)** Immunofluorescence of Villin in an F4 human tubuloid. **(K)** Crude tubuloid showing a positive staining for Calbindin. **(L)** (Crude tubuloid) and **(M)** (F4 tubuloid), immunofluorescence for AQP3, CADH1 and cytokeratin. Tubuloids were obtained from n = 7 independent kidney tissue donors, and each condition was repeated in duplicate. **(N)** Expression of tubular markers in human tubuloids. Black box: liver tissue; Blue box: F4 tubuloids; Yellow box: Crude tubuloids. *P < 0.05, **P < 0.001, ****P < 0.0001. Data are represented as mean ± SEM. For the qPCR analysis, there were n = 3 independent liver tissue donors (as controls), n = 7 independent kidney tissue donors for the F4 tubuloids and n = 4 independent kidney tissue donors for the Crude tubuloids (matched with the F4 tubuloids’ donors). Triplicates were performed for each sample and condition.

To increase tubular cell purity from the whole kidney sample we included a tubular cell enrichment step as previously described by Vinay et al. [11] The digested tissue was sequentially sieved through 320 μm and 150 µm sieves and then resuspended in Matrigel^®^ (Corning, NY, United States) and plated to obtain tubuloids (protocol A, Crude tubuloids). We performed a second protocol (protocol B, F4 tubuloids) in which the tissue underwent a second separation process by centrifugation with 45% Percoll^®^ (Sigma-Aldrich, Barcelona, Spain) (Figure 1A). See the Supplementary Methods section, Human tubuloid culture.

### Rat Tubuloid Culture

Rat kidney tissue for rat tubuloid culture was collected from 3 healthy 3-month-old Lewis rats (LEW/Han®Hsd, Envigo^®^, Barcelona, Spain). Tubuloid isolation and culture were performed following the same methods as those described for human tubuloids. See the Supplementary Methods section, Rat tubuloid culture.

### GFP Tubuloid Development

To track tubuloids in delivery studies, GFP-expressing rat tubuloids were obtained from a GFP rat strain (CAG-GFP rat line, genOway, Paris, France) with constitutive GFP expression. The isolation and culture method performed for GFP rat tubuloids from this strain was similar to the one previously detailed. Both protocols (F4 and Crude) were performed. GFP fluorescence was confirmed immediately after plating with a fluorescence microscope.

### Subcapsular and Intracortical Tubuloid Injection *In Vivo*

GFP-expressing rat tubuloids were harvested and resuspended in pure Matrigel^®^ (Corning, NY, United States) without previous disruption. A Lewis rat was selected as a host (LEW/Han®Hsd, Envigo^®^, Barcelona, Spain). The animal was anesthetized with isoflurane inhalation, and a mid-abdominal incision was performed to expose the abdominal organs. Approximately 1·10^6^ cells were injected into the left kidney: two injections were performed (one subcapsular in the upper kidney pole and one intracortical in the lower pole) with a 25G needle. The rat was euthanized 1 day after the procedure and both native kidneys were collected for analysis.

### Tubuloid Infusion During Rat Kidney Normothermic Perfusion

Rat donor organ procurement was performed under anesthesia with isoflurane (IsoVet^®^, Braun, Barcelona, Spain) as previously described [12]. Rat kidney NMP was performed following the protocol published elsewhere [13].

GFP-expressing rat tubuloids were harvested and resuspended in pure Matrigel^®^ (Corning, NY, United States) with previous mechanical disaggregation and then were administered in the perfusate through the arterial line. The kidney grafts were maintained under NMP for 1 h. For each donor, one kidney graft was perfused and treated with tubuloids, while the contralateral one was perfused but no tubuloids were administered. We tested two doses of tubuloids: 1·10^6^ cells/g (high dose, n = 4 kidneys) and 3·10^5^ cells/g (low dose, n = 3 kidneys). See the Supplementary Methods section, Tubuloid infusion during rat kidney normothermic perfusion.

### Kidney Transplantation in Rats

Inbred 3-month-old Lewis rats were used as recipients for kidney grafts preserved in NMP. Since the CAG-eGFP rat strain had a Sprague-Dawley background, a short dose of tacrolimus was initiated to prevent a potential rejection response against GFP tubuloids. Thus, 0.5 mg/kg of tacrolimus (Modigraf^®^, Astellas, Barcelona, Spain) was administered intramuscularly to the Lewis recipient for four consecutive days (−1, 0, +1 and +2 relative to transplantation–day 0) [14]. Kidney transplants were performed as previously described by our research group [12]. See the Supplementary Methods section, Kidney transplantation in rats.

### Tubuloid Infusion During Human Kidney Normothermic Perfusion

Human kidneys were obtained from a patient who was declared a potential donor and the organs were evaluated according to our center’s policy. Kidneys were only accepted for research purposes after the decision to be discarded according to clinical criteria. The study protocol was reviewed and approved by the Research Ethics Committee of our center (Comité de Ética de la Investigación con medicamentos, CEIm). Written informed consent to participate in this study was provided by the patient’s relatives.

Normothermic Perfusion was performed using the ARK Kidney device from Ebers Medical^®^ as previously described by our research group [10]. Both kidneys from the same donor were perfused simultaneously, and GFP-rat tubuloids (1·10^6^ cells/g) were administered to one of them through the arterial line once the organ was stable. Both kidneys were maintained for 6 h.

For more detailed information regarding these Methods, see the Supplementary Material section.

### Statistical Analysis

Statistical analysis was performed using a Student’s t-test or ANOVA test, as appropriate. The data were represented in graphs using GraphPad Prism version 9.0.0 for Mac (GraphPad Software, San Diego, California, USA). A P-value <0.05 was considered significant.

## Results

### Crude and F4 Human Kidney Tubuloids Show Typical Tubular Polarity and Specific Markers of Different Nephron Segments

Human tubuloids typically developed approximately 7 days after plating. Optical microscopy (Figures 1B,C) and Hematoxylin and Eosin staining (Figure 1D) showed that fully developed tubuloids acquired the typical morphology of a polarized epithelium with a single cell layer and a spheroid shape, which was usually observed after 3-4 passages. No significant differences in culture or development were observed between Crude and F4 tubuloids.

To assess the tubular epithelial nature of the obtained tubuloids, a specific immunostaining was performed using a specific antibody against PAX-8 (Figure 1E). After assessing the tubular nature of the tubuloids, we further analyzed the presence of a typical tubular polarization in the epithelium: ZO-1 (a tight junction tubular protein) was predominantly situated on the lateral and apical sides (Figure 1F). Polarization was also evidenced by combining immunofluorescence of pan-cytokeratin (basolateral) and acetylated tubulin (apical) (Figures 1G,H). Integrin **α**6 (which maintains the integrity of the kidney tubular epithelium) was mostly expressed on the basolateral side of the tubuloids’ cells (Figure 1I).

Immunostaining for Villin 1 (apical) identified proximal tubular cells (Figure 1J). Distal cells were identified by positive immunostaining for Calbindin-1 (an intracellular calcium-binding protein that is localized in the distal tubule cells) on the apical side (Figure 1K). E-cadherin (CADH1), a basolateral marker for the adherens junctions, is typically present in distal and collecting duct cells. However, in collecting duct cells, CADH1 is usually expressed with AQP3 (a channel expressed only in collecting duct cells). We identified the presence of CADH1-positive tubuloids, although no concomitant expression of AQP3 was evident, thus suggesting a distal nature of these tubular cells (Figures 1L,M). Remarkably, tubuloids expressing a marker for a specific nephron segment did not express markers for other segments, indicating that a predominant tubular cell type constitutes the tubuloid. These findings were observed in Crude and F4 tubuloids.

Proximal gene ABCC4 expression was higher when compared to the liver, along with SLC12A1 (loop of Henle), SLC12A3 and SLC41A3 (distal tubule). AQP3 (collecting duct) was not significantly expressed in human tubuloids. F4 tubuloids exhibited higher expression of all tubular markers compared to Crude tubuloids (Figure 1N).

### Expanding F4 Human Kidney Tubuloids Consist of Cells With Polarized Proximal and Distal Tubular Phenotypes, Compared to Crude Tubuloids

To further investigate the different cell types present in the human tubuloids developed through our two methods, we performed single-cell RNA (scRNA) sequencing on Crude Tubuloids (n = 4, 49442 cells, Passages 3 and 4) and F4 Tubuloids (n = 3, 29998 cells, Passages 3 and 4). Clustering analysis resulted in the identification of 2 distinct clusters in both Crude and F4 tubuloids (Cluster 0 and Cluster 1) (see Supplementary Table S1).

**FIGURE 2:**
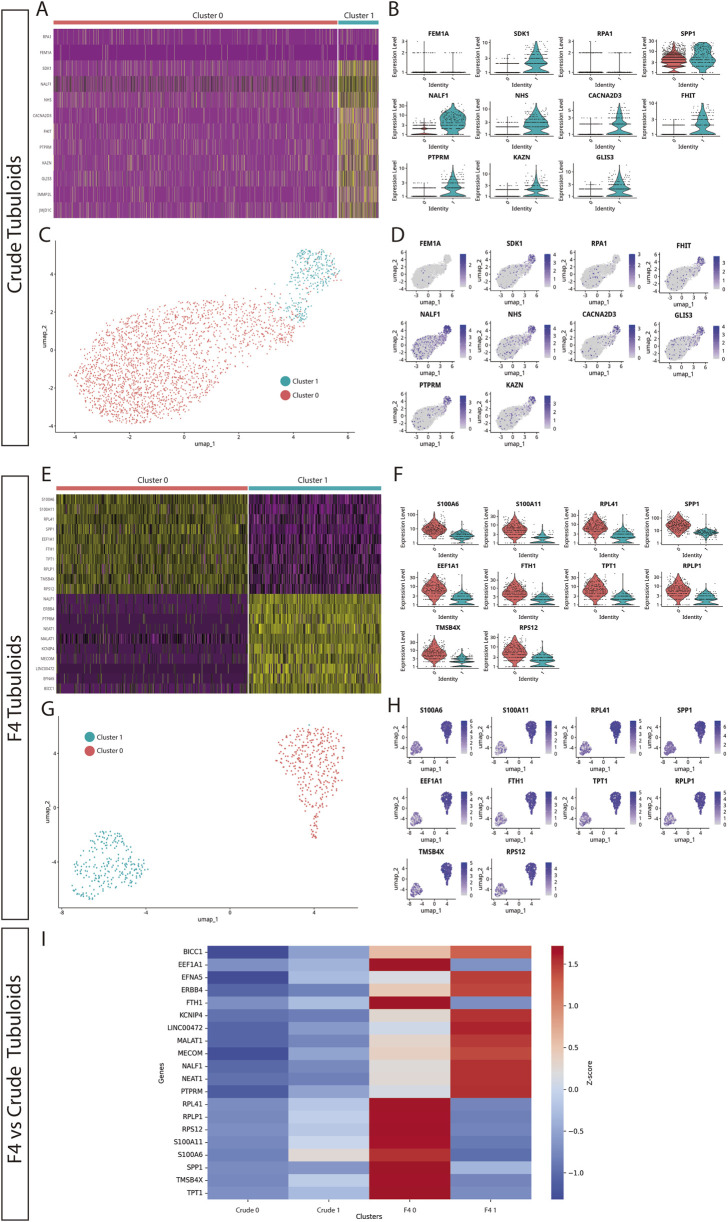
Expanding F4 human kidney tubuloids consist of cells with polarized proximal and distal tubular phenotypes compared to Crude tubuloids. **(A)** Heatmap showing the gene expression patterns of two cell clusters in Crude tubuloids. **(B)** Differentially expressed genes between the identified clusters in Crude tubuloids. **(C)** Uniform manifold approximation and projection (UMAP) plot and clustering of single-cell RNA sequencing data of Crude tubuloids (n = 4, 49442 cells, Passages 3 and 4). **(D)** Feature plot for the differentially expressed genes in Crude tubuloids. **(E)** Heatmap showing the gene expression pattern of two cell clusters in F4 tubuloids. **(F)** Differentially expressed genes between the identified clusters in F4 tubuloids. **(G)** UMAP plot and clustering of single-cell RNA sequencing data of F4 tubuloids (n = 3, 29998 cells, Passages 3 and 4). **(H)** Feature plot for differentially expressed genes in F4 tubuloids. **(I)** Heatmap of normalized Z-scores showing expression of proximal and distal tubular markers across Crude and F4 tubuloids. See also Supplementary Table S1.

For Crude tubuloids both clusters exhibited an overlapping of tubular cell marker expression: Cluster 1 displayed a predominant proximal tubular cell signature (marked by the expression of NHS, FHIT and PTPRM [15, 16]), along with a minor expression of distal tubular markers (represented by CACNA2D3 [15, 16]). This pattern was lacking in Cluster 0, although reduced expression of almost every tested marker was also present. This minor expression of segment-specific genes may suggest a population with a more progenitor status. In both clusters, a small population of cells with a collecting duct signature (marked by NALF1 and RPA1 [15, 17]) was identified (Figures 2A–D).

Two cell clusters with significantly different expression signatures were also identified in F4 tubuloids: Cluster 0 displayed a predominant proximal tubular cell signature (marked by FTH, SPP1, TPT1 and RPLP1 [15, 16]), whereas Cluster 1 mostly displayed a predominant distal tubular cell signature (marked especially by KCNIP4 and NEAT1 [15, 16]) (Figures 2E–H). Notably, both clusters showed clear separation of gene expression for specific-segment genes, suggesting a higher degree of differentiation than Crude tubuloids. Cluster 0 also showed marked expression of S100A6, which is involved in calcium management and has been associated with tubuloid-derived progenitors from the thick ascending limb [3]. Furthermore, F4 tubuloids displayed high expression of genes involved in protein synthesis and cell proliferation (RPS12, MECOM, BICC1 [15, 16]), thus indicating a high proliferative status.

When gene expression of tubular markers from both culture protocols was compared through Z-score normalization, we observed that Crude tubuloids exhibited negative Z-scores for the majority of the analyzed genes, indicating low relative expression compared to the overall mean across conditions. This was especially evident in Crude Cluster 0, while Cluster 1 displayed higher expression levels. In contrast, F4 tubuloids were associated with predominantly positive Z-scores, suggesting higher expression levels of the analyzed genes. Furthermore, a polarized expression of proximal (F4 Cluster 0) or distal (F4 Cluster 1) tubular markers was observed. These differential patterns indicate that the F4 culture method may provide an environment more conducive to gene expression changes associated with differentiation (Figure 2I).

### Tubuloids Can Be Obtained From Adult Rat Kidney Tissue and Serve as a New *In Vitro* Platform for Studying Kidney Diseases

**FIGURE 3:**
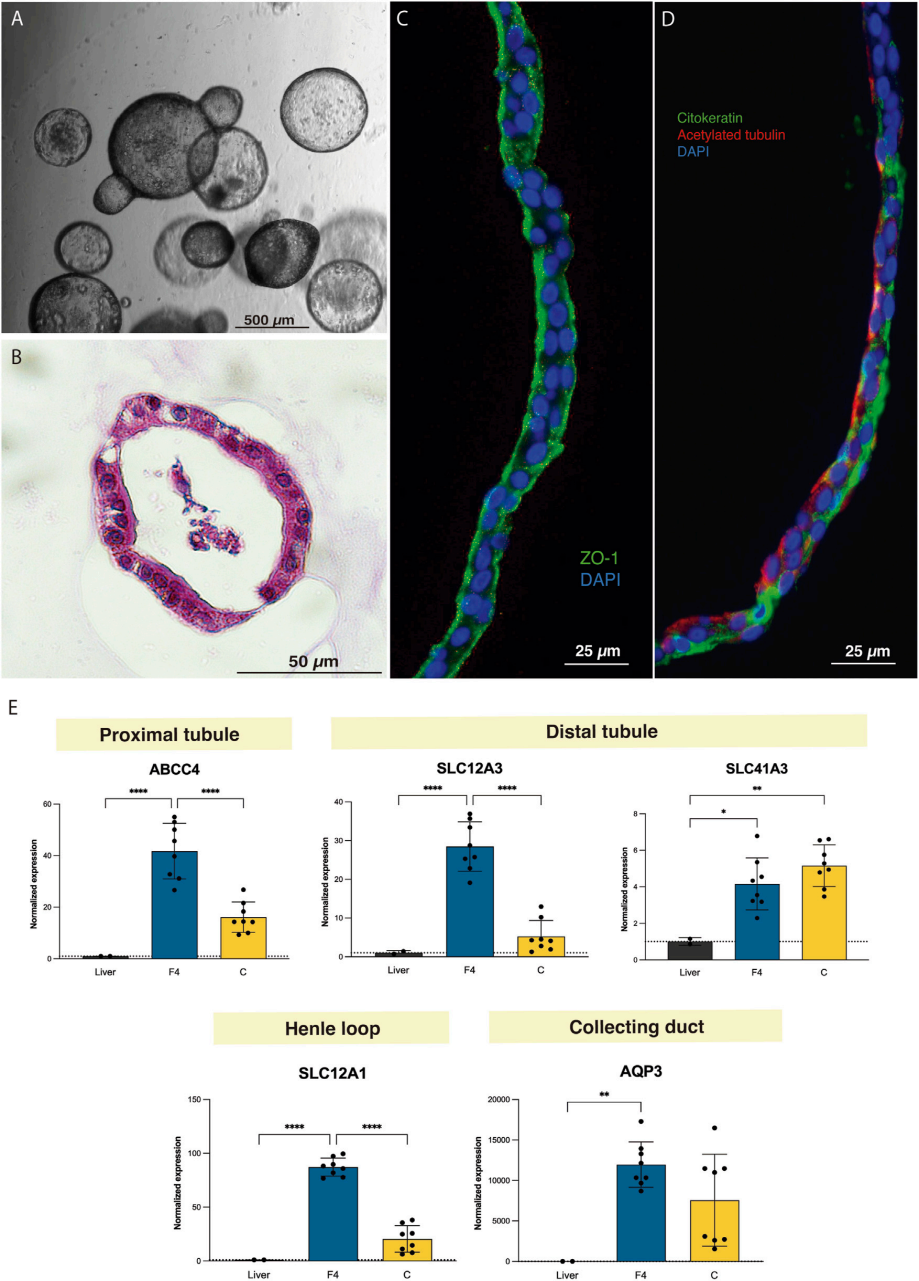
Tubuloids can be obtained from adult rat kidney tissue. **(A)** Optical microscopy image of rat tubuloids showing a uniform spherical morphology. **(B)** Paraffin sections with HE staining of rat tubuloids. **(C,D)** Confocal images of rat tubuloids showing epithelial polarization similar to that of human tubuloids for ZO-1 **(C)**, for cytokeratin and acetylated tubulin **(D)**. **(E)** Expression analysis of rat tubuloids for different tubular genes. Black box: liver tissue; Blue box: F4 rat tubuloids. Yellow Box: Crude rat tubuloids. *P < 0.05, **P < 0.001, ****P < 0.0001. Data are represented as mean ± SEM. Rat tubuloids were obtained from n = 8 independent Lewis rats, seeded in duplicate to obtain matched F4 and Crude tubuloids. For the qPCR analysis, liver tissue was obtained from 2 independent Lewis rats. Each sample was analyzed in triplicate for each condition.

Rat tubuloids developed after 10–14 days and acquired a uniform spherical morphology, which was preserved for up to 17 passages (Figures 3A,B). Immunofluorescence assessment evidenced tubular epithelial polarity, similar to that of human tubuloids (Figures 3C,D).

Gene expression analysis for segment-specific markers evidenced the expression of proximal, Loop of Henle and distal tubule markers, especially in F4 rat tubuloids. In contrast to human tubuloids, rat tubuloids expressed the collecting duct marker AQP3 (Figure 3E).

### Kidney *Ex Vivo* Normothermic Perfusion Is an Efficient Strategy for Delivering Tubuloids to the Kidney Parenchyma

**FIGURE 4:**
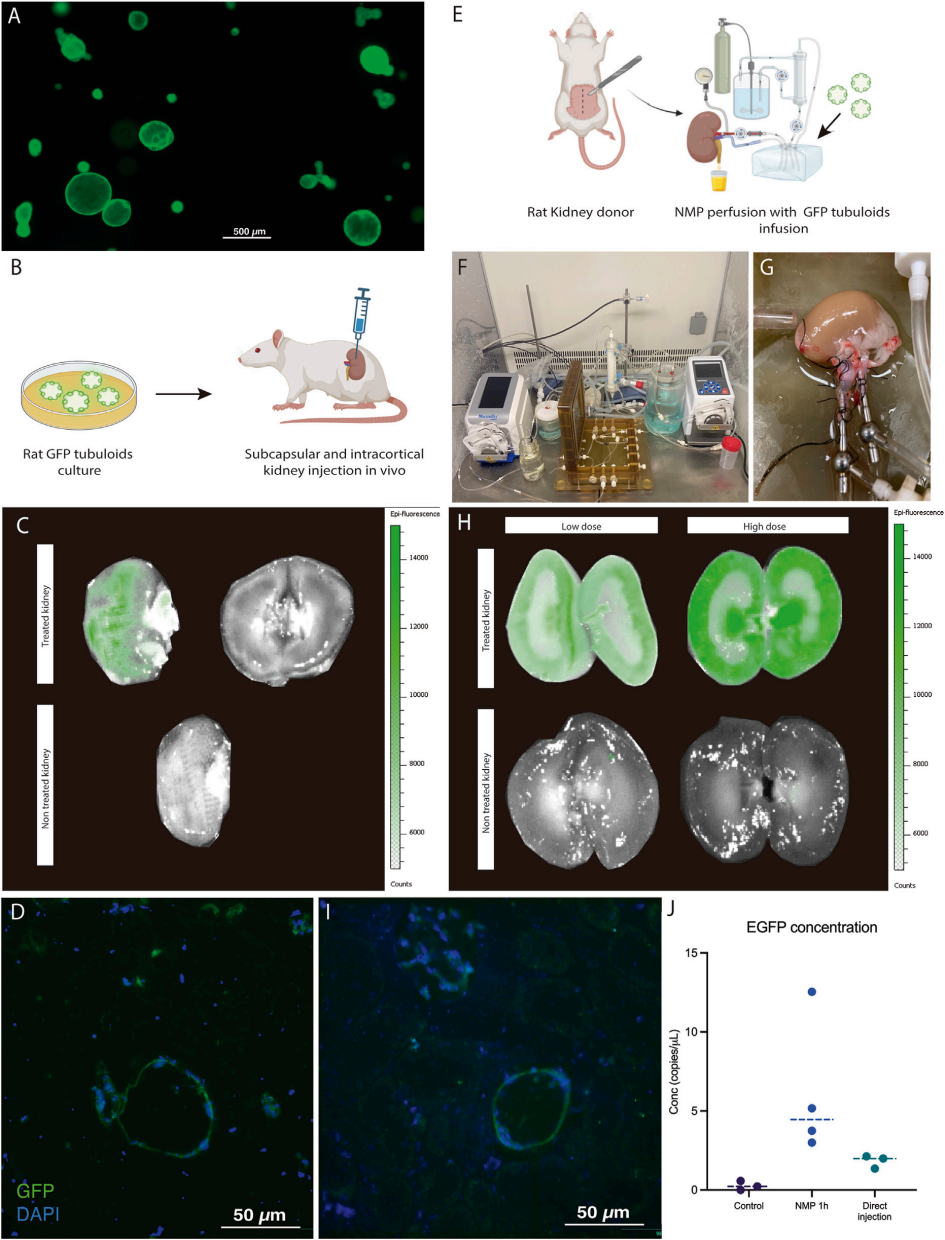
Kidney *ex vivo* normothermic perfusion is an efficient strategy for delivering tubuloids to the kidney parenchyma compared to *in vivo* administration. **(A)** Fluorescence Optical Microscopy image of rat tubuloids obtained from CAG-eGFP rat kidney tissue (obtained from n = 5 independent rat kidney donors). **(B)** Scheme of *in vivo* injection of GFP tubuloids in a rat kidney host. **(C)** Epifluorescence analysis of the GFP signal in an injected kidney with GFP tubuloids (n = 3 injected rats). The image shows the outer and internal parenchyma of one representative case of an *in vivo* injected kidney, along with a control one (no injection). **(D)** Representative image of a GFP tubuloid observed in the kidney parenchyma after subcapsular injection (n = 3 injected rats, each immunofluorescence analysis was performed in duplicate). **(E)** Scheme of the GFP tubuloid injection during kidney normothermic preservation. **(F,G)** Detail of the normothermic system for rat kidney normothermic preservation. **(H)** IVI images of GFP fluorescence in perfused and treated kidneys with high (n = 4 kidneys from independent Lewis donors) and low doses (n = 3 kidneys from independent Lewis donors) of tubuloids and perfused, non-treated kidney grafts (n = 3 kidneys from independent Lewis donors). **(I)** Representative immunofluorescence image for GFP showing a GFP tubuloid in the kidney cortex after normothermic perfusion and tubuloid infusion (high dose, n = 4 kidneys from independent donors, each condition performed in duplicate). **(J)** Determination of dPCR eGFP copies for kidneys perfused with GFP tubuloids and those in which direct injection was performed. Data are represented as individual values and the median. Control, n = 3 perfused, non-treated kidneys. NMP 1h, n = 4 perfused and treated kidneys, high dose. Direct injection, n = 3 injected rats. For dPCR, each sample was analyzed in duplicate for eGFP expression. Epifluorescence and immunofluorescence images were acquired using the same acquisition and detection settings, and a protocol was performed to mitigate kidney autofluorescence. The GFP signal was corrected for background.

Epifluorescence assessment of the *in vivo* injected kidney with the IVIS^®^ Lumina device evidenced a positive GFP signal in the injected kidney compared to the contralateral kidney (without treatment). This signal was localized to the surface of the kidney where the injections were performed (Figures 4A–C). No evident signal was detected in the internal part of the kidney. Immunofluorescence analysis showed the presence of GFP-positive structures measuring 150–200 μm in diameter localized in the injection area (Figure 4D).

The second strategy consisted of infusing GFP tubuloids into the perfusate during *ex vivo* normothermic perfusion of the kidney graft (Figures 4E–G). We tested two cell doses to assess a potential correlation with the fluorescent signal observed in the IVIs analysis (high dose: 1·10^6^, and low dose: 3·10^5^ cells/g). Tubuloid infusion was usually followed by a temporary increase in vascular resistance (see Supplementary Figure S2). The IVIs study evidenced a diffuse GFP signal primarily in the kidney cortex, with no significant signal in the perfused, non-treated kidney (Figure 4H). The registered fluorescence signal was higher and acquired a more diffuse distribution than with *in vivo* tubuloid injection (Figures 4C,H). When testing a lower dose of tubuloids, a similar pattern, although with a decreased signal, was identified in the treated kidney graft (Figure 4H). Immunofluorescence analysis showed the presence of GFP-positive structures measuring 30–50 μm in diameter within the cortical kidney parenchyma (Figure 4I).

Kidney grafts treated with tubuloids during NMP presented the highest number of EGFP copies, with a concentration that ranged from 3 to 12 copies/μL. In vivo-injected kidney grafts showed fewer EGFP copies (ranging from 2 to 3 copies/μL), although this was higher than the controls (Figure 4J).

### Tubuloids Delivered to the Kidney During NMP Integrate Into the Host Kidney Parenchyma and Form New Tubular Structures

**FIGURE 5:**
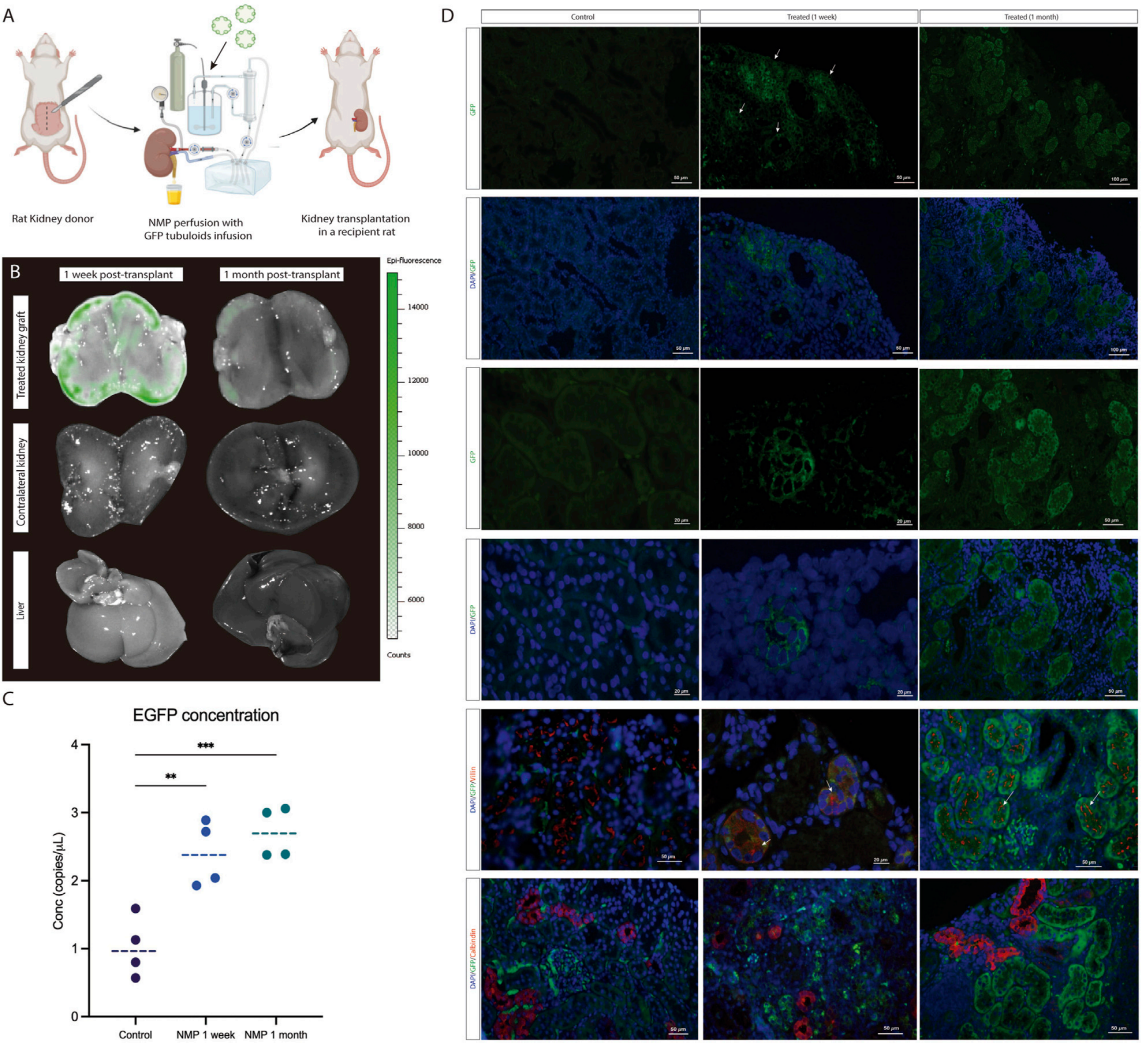
Tubuloids delivered to the kidney during NMP integrate into the host kidney parenchyma and form new tubular structures. **(A)** Scheme of the GFP tubuloid injection during human kidney normothermic preservation and transplantation. **(B)** IVI images of GFP fluorescence in the treated kidney graft, native kidney, and liver. **(C)** dPCR eGFP copy number determination for kidneys perfused with and without GFP tubuloids. **(D)** Immunofluorescence images for GFP, Villin and Calbindin-1 of the control and treated kidneys showing GFP and Villin-expressing cells (white arrows) constituting new tubuloids but also integrated into the host tubular epithelium after normothermic perfusion and tubuloid transplantation. The data are represented as individual values and medians. *P < 0.05. **P < 0.01. ***P < 0.001. N = 4 transplanted rats per group (control, 1 week and 1 month). For the immunofluorescence and dPCR studies, each sample was analyzed in duplicate. Epifluorescence and immunofluorescence images were acquired using the same acquisition and detection settings, and a protocol was performed to mitigate kidney autofluorescence. The GFP signal was corrected for background.

One week and one month after kidney NMP and tubuloid infusion (Supplementary Figures S1, S2; Figures 5A,B). Notably, GFP fluorescence decreased relative to the kidney graft immediately after NMP and showed a further decline after 1 month. No signal was detected either in the contralateral native kidney or the liver at the analyzed time points (Figure 5B). DdPCR analysis of GFP copies evidenced a higher concentration in the treated grafts compared to the controls, a difference that persisted at 1 month (Figure 5C). Immunofluorescence for GFP in the kidney graft showed the presence of GFP-expressing cells in the tubular compartment (Figure 5D). Remarkably, these cells formed new, fully-developed tubules in the host kidney parenchyma and were also found to integrate the pre-existing epithelium of host tubules. These newly developed tubules showed positive Villin expression, while Calbindin-1 expression was not observed, thus suggesting a proximal phenotype with no formation of distal tubules (Figure 5D).

### *Ex Vivo* Normothermic Perfusion Efficiently Delivers Tubuloids to a Human Kidney Graft

The kidney donor was a 56-year-old man with no relevant medical history who experienced a cardiac arrest with no recovery after 90 min of basic and advanced life support. Once identified, organ retrieval was immediately started as an Uncontrolled Donor after Cardiac Death (uDCD). Serum creatinine at the time of donation was 1.5 mg/dL, although poor perfusion of both kidneys was observed during bench surgery; therefore, the kidneys were discarded for transplantation.

**FIGURE 6:**
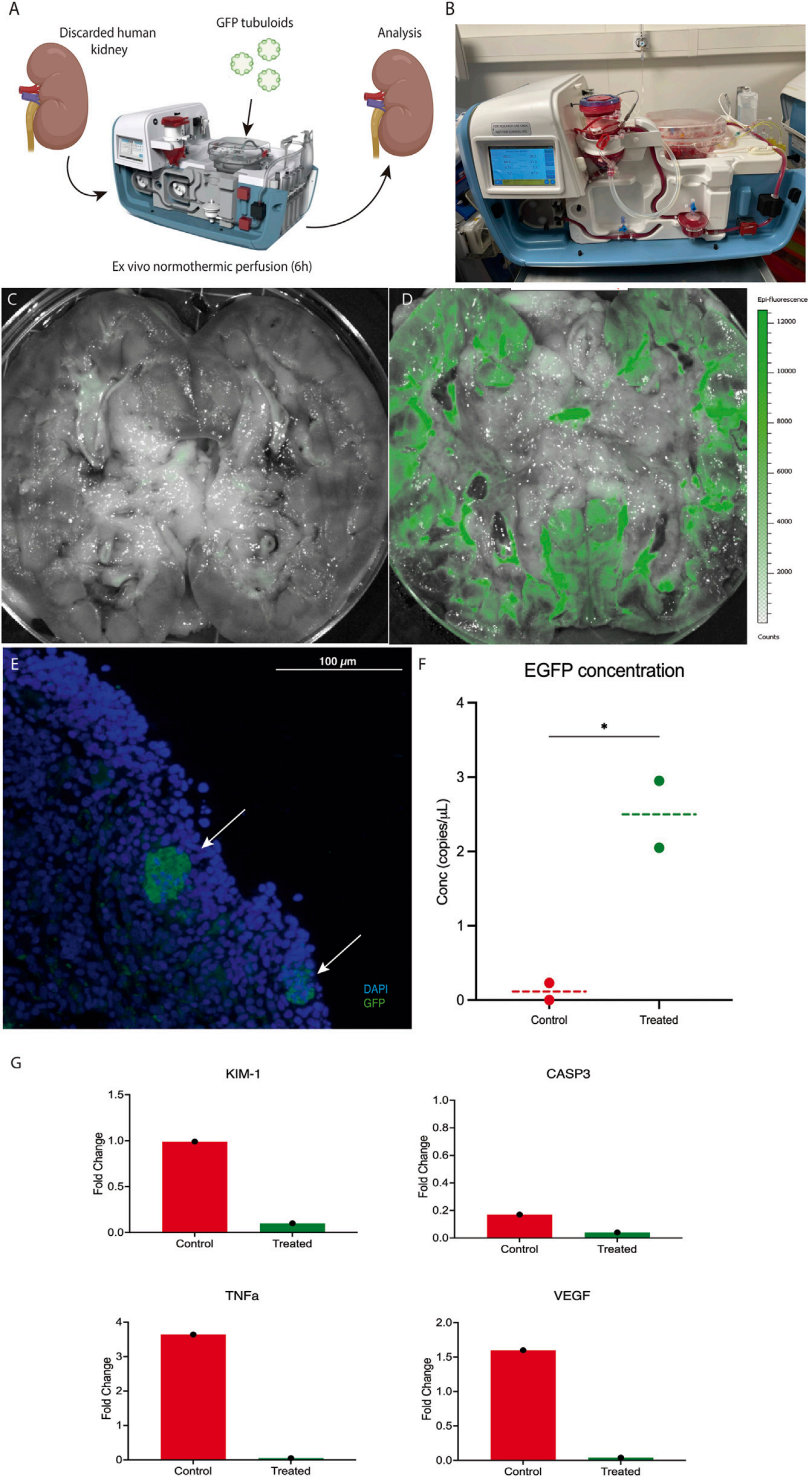
*Ex vivo* normothermic perfusion efficiently delivers tubuloids to a human kidney graft. **(A)** Scheme of the GFP tubuloid injection during human kidney normothermic preservation. **(B)** Details of the normothermic system for human kidney normothermic preservation. **(C,D)** IVI images of GFP fluorescence in perfused kidneys without **(C)** and with GFP tubuloids **(D)**. **(E)** Representative immunofluorescence image for GFP showing GFP tubuloids (white arrows) in a human kidney after normothermic perfusion and tubuloid infusion. **(F)** dPCR eGFP copy number determination for treated and non-treated human kidneys with GFP tubuloids during NMP. **(G)** Kidney injury and inflammatory markers during normothermic perfusion in treated and control human kidneys. Gene expression has been represented as the fold change at 6 h of perfusion from time 0. KIM-1, Kidney Injury Marker 1; CASP3, Caspase 3; TNFa, Tumor Necrosis Factor alfa; VEGF, Vascular Endothelial Growth Factor. N = 1 kidney per group (control and treated). Each sample was run in triplicate. Each dot represents a random sample from the same kidney parenchyma. The line represents the median. N = 1 kidney per group (control and treated). Each sample was processed in duplicate for dPCR and immunofluorescence analysis. Epifluorescence and immunofluorescence images were acquired using the same acquisition and detection settings, and a protocol was performed to mitigate kidney autofluorescence. The GFP signal was corrected for background.

Both kidneys were simultaneously cannulated and connected to the Ebers^®^ NMP perfusion device (Figures 6A,B). After stabilization, the GFP tubuloids were mechanically disaggregated into 50 μm structures and then administered into one of the kidneys through the arterial cannula at a dose of 1·10^6^ cells/g. The kidneys were perfused for 6 h.

After 6 h, an epifluorescence assessment showed a positive and diffuse GFP fluorescence sign in the perfused, treated kidney, primarily located in the kidney cortex, with no significant signal in the perfused, non-treated kidney (Figures 6C,D). Immunofluorescence assessment evidenced the presence of 20 -50 μm GFP-positive cell aggregates in the cortical graft of the treated kidney (Figure 6E). Quantification of GFP copies in kidney tissue showed a higher concentration in the treated graft compared to the control (2.5 vs. 0.1 copies/μL, respectively) (Figure 6F). Hemodynamics during perfusion are shown in Supplementary Figure S3. From a functional perspective, during NMP, the tubuloid-treated kidney showed higher urine output compared to the control (Supplementary Figure S3I). In addition, the expression levels of key markers of kidney injury (KIM-1), apoptosis (CASP3), and inflammation (VEGF, TNFα) were lower in the treated organ after 6 h of perfusion, suggesting a reduction in inflammatory damage during NMP (Figure 6G).

## Discussion

Although kidney transplantation is the treatment of choice for patients with End Stage Kidney Disease (ESKD), access to kidney grafts is severely limited due to the scarcity of available organs for transplantation [18]. Thus, new therapies that increase the donor pool are urgently needed. In this sense, regenerative medicine encompasses a broad spectrum of strategies with the potential to overcome organ scarcity through kidney regeneration [7, 19–21]. We have proposed two modified protocols for obtaining human tubuloids from human kidney tissue by adding a tissue sieving step to isolate tubular cells before culture. The F4 method also involved a Percoll gradient after sieving to obtain a purer tubular proximal cell population.

The resulting tubuloids from both methods expressed segment-specific markers corresponding to the proximal tubule, loop of Henle, and distal tubule, but lacked markers of the collecting duct. These findings were consistent with the immunofluorescence analysis, in which no expression of AQP3 was evidenced, thus suggesting that collecting duct cells were underrepresented [2, 22, 23]. Remarkably, when assessing the presence of different nephron segments by immunofluorescence, each tubuloid (even those with the same origin) tended to consist of cells with a predominant nephron segment phenotype, a feature that has been previously described [22, 24–26].

Single-cell analysis of both culture methods showed mixed expression of proximal, tubular and collecting duct markers, which suggests a mixture of different cell populations. Notably, the absence of a specific expression of nephron markers suggests a cell population in Crude tubuloids with a more progenitor status [3]. In contrast, F4 tubuloids consisted of two cell clusters with two clearly differentiated phenotypes: one cluster was integrated by a cell population with high expression of proximal tubular markers, while the other one expressed mostly distal tubular markers. A clear expression pattern of polarization was observed in F4 tubuloids compared to Crude tubuloids, in which cell clusters overlapped. These results may be explained by the origin of the Crude tubuloids, since in this case all tubular cell types were cultured. This mixture of different tubular cells when culturing Crude tubuloids may lead to a higher number of tubular cell phenotypes with less tubuloid cell nephron-specific differentiation [3, 27].

To date, the majority of studies have relied on obtaining human tubuloids, which have received significant attention [1–3]. Furthermore, the development of mice-derived tubuloids has recently been described as a new model for studying kidney disease [26, 28–30]. Nevertheless, rats have been demonstrated to be a more reliable and advantageous model for kidney disease research than mice, as they have larger organs (facilitating surgical procedures and sample collection and allowing for more precise studies), their renal physiology and pathology more closely resemble that of humans (improving the relevance of experimental findings) and they also have slower disease progression, enabling long-term kidney function and regeneration studies. Thus, to provide a new tool for studying kidney disease, we developed rat-derived tubuloids, following the same modified protocols but using specific rat growth factors. In contrast to human tubuloids, healthy rat-derived tubuloids developed later and expressed markers of every nephron segment, even from the collecting duct. These findings may suggest a higher differentiation capacity of rat tubular cells in this specific environment, positioning this model as an available tool for further kidney disease research [1, 2, 22]. In our study, tubuloids were obtained from kidney tissue. Nevertheless, the use of urine as a source of tubular cells for tubuloid culture has also been described, and it indeed represents a promising, non-invasive method for tubuloid culture [2, 31]. However, obtaining tubular cells from urine poses several challenges. In healthy individuals, the number of viable tubular cells present in urine is typically low, which may significantly reduce the efficiency of this approach. Consequently, large volumes of urine are required to isolate a sufficient number of cells for successful tubuloid generation. Additionally, there is a high risk of contamination during primary culture, which further compromises the efficiency of this method [32, 33]. Therefore, urine was not used as a cell source in our study, especially in our rat models, where collecting large volumes of sterile urine is even more difficult.

However, urine from individuals with kidney disease may contain a higher number of tubular cells. This is likely due to increased cell shedding and endogenous tubular regeneration, as observed during acute tubular necrosis [32]. In these cases, we believe that tubuloid generation from urine could be a more viable and efficient option, offering a non-invasive source of tubuloids for potential use in personalized therapies targeting the donor’s kidney disease [31].

In summary, we encountered significant technical challenges when using urine as a source of tubular cells, mainly due to the low yield of cells and the risk of contamination. However, we recognize the promising potential of urine as a future non-invasive source of tubular cells for tubuloid development and personalized medicine.

The proliferation capacity of tubuloids has the potential ability to regenerate kidney tissue and accelerate tissue repair in cases of kidney injury [34], although an optimal delivery and engraftment route to the kidney tissue has yet to be established, especially when considering clinical applicability [4, 5, 35–39]. Recently, NMP in kidney organ transplantation has emerged as a promising platform for long-term organ preservation and treatment testing while the organ is physiologically preserved and monitored [10, 40, 41]. One of this platform’s most relevant characteristics is its fast translatability to clinical practice, since it allows for the testing of innovative treatments in human organs and the monitoring of their effects in a safe environment [7, 42, 43].

In our study, epifluorescence was more intense and diffuse in those grafts treated during NMP compared to those injected *in vivo*, in which the signal was weaker and localized to the injection area. A weaker signal was also observed when a lower dose of tubuloids was administered during NMP, suggesting a signal-cell dose relationship. Our findings were also reinforced by GFP copy quantification, since the NMP kidneys showed copy numbers of up to 12- and 5-fold higher compared to controls and injected kidneys, respectively.

In our study, at 1 month, GFP-expressing cells were still detected, either constituting either fully developed tubules or the tubular epithelium alongside host tubular cells. Similar results were reported by Sampaziotis et al. [6] in cholangiocyte-derived organoids. Our results further evidenced the engraftment capacity of tubuloids not only to integrate into a pre-existing tubular epithelium but also to generate new fully tubular structures among the host kidney parenchyma with a predominant proximal phenotype. No signal was identified in either the native kidneys or the liver after 1 month, suggesting that our approach significantly increases specific delivery to the kidney while minimizing cell leakage to non-target organs. In our scenario, cell aggregates of 20–25 μm were injected, and considering the fact that glomerular capillaries are only 6–10 μm, this may lead to blockages and severe disruption of perfusion. Despite this, we observed only a transient increase in vascular resistance during infusion, with no signs of long-term hemodynamic compromise, vascular occlusion, or histological evidence of microvascular damage. We hypothesize that this observation is related to an increase in glomeruli and endothelial permeability associated with ischemia-reperfusion injury, thus allowing the transition of cellular aggregates [44–46]. Overall, post-infusion biodistribution analysis revealed no detectable signal in non-target organs, including the native kidneys and liver, 1 month after cell delivery. This indicates minimal off-target migration and suggests that our approach achieves the specific and localized retention of therapeutic cells within the injured organ. Nonetheless, the risk of vascular obstruction or ectopic engraftment cannot be entirely excluded, particularly in models without prior injury or in clinical scenarios with variable vascular integrity. Therefore, future studies should include detailed histological and functional assessments of both target and non-target organs in larger animal models to confirm the long-term safety of this strategy.

When moving to a closer clinical scenario using a discarded human kidney, we observed that, after 6 h of perfusion, a diffuse GFP signal in the cortex was detected. The presence of tubuloids was also confirmed by observing GFP-positive structures in the kidney parenchyma and a 3-fold concentration of GFP copies compared to the control, perfused kidney. Our results are in line with those published in 2020 by Thompson et al [43]. In addition, after 6 h of normothermic machine perfusion, the treated kidney graft exhibited lower expression levels of key markers associated with kidney injury (KIM-1), apoptosis (CASP3), and inflammation (VEGF, TNFα) compared to the control. This molecular profile was accompanied by a modest but consistent increase in urine output in the treated organ. Together, these findings suggest that the infused tubuloids may exert a protective effect, potentially mitigating ischemia-reperfusion injury by modulating early inflammatory and apoptotic responses. These functional and molecular improvements highlight the therapeutic promise of tubuloid-based interventions in renal graft preservation and recovery.

Our study has some limitations. Although it suggests that tubuloids can be efficiently delivered and integrated into a host kidney, we did not assess their positive impact on kidney function since the transplantation model was performed to assess biodistribution and engraftment, and no native nephrectomy was performed on the rat recipient. In the human kidney analysis, although we observed a potential positive effect of tubuloid infusion on expression parameters, only one case was performed, so solid conclusions cannot be drawn. Thus, further studies to assess the potential benefits of tubuloid implantation in kidney conditioning and improvement are needed.

In conclusion, our work reinforces the role of tubuloids as a key point for studying kidney diseases and developing new treatments, in addition to promoting kidney tubular regeneration. Moreover, our study provides evidence of the engraftment ability of tubuloids and establishes NMP as an efficient technique for regenerative cell therapy that overcomes off-target delivery to other organs. Since NMP is a platform that has already been developed for solid human organs in clinical practice, this strategy could be a promising approach for kidney conditioning and regeneration with fast translatability to clinical practice. Future research on regenerative strategies is necessary to exponentially expand knowledge of tissue regeneration and rapidly translate these therapeutic advances into clinical practice.

## Data Availability

The datasets presented in this study can be found in online repositories. The names of the repository/repositories and accession number(s) can be found below: https://www.ncbi.nlm.nih.gov/, PRJNA1153012.
